# Editorial: Diabetes, transplantation and regenerative medicine

**DOI:** 10.3389/fcdhc.2024.1388904

**Published:** 2024-04-22

**Authors:** Alessandro Mattina, David Baidal, Braulio Marfil-Garza, Margherita Occhipinti, Valeria Sordi

**Affiliations:** ^1^ Diabetes Service, IRCCS-Istituto Mediterraneo per i Trapianti e Terapie ad alta specializzazione (ISMETT), University of Pittsburgh Medical Center (UPMC), Palermo, Italy; ^2^ Diabetes Research Institute (DRI) and Clinical Cell Transplant Program, University of Miami Miller School of Medicine, Miami, FL, United States; ^3^ The Institute for Obesity Research, Tecnologico de Monterrey, Monterrey, NL, Mexico; ^4^ Diabetes Unit, Versilia Hospital, Lucca, Italy; ^5^ Diabetes Research Institute, IRCCS San Raffaele Hospital, Milan, Italy

**Keywords:** diabetes mellitus, transplantation, post-transplant diabetes mellitus (PTDM), beta-cell replacement therapy, pancreas transplantation, islet transplantation, stem cell-based therapy, type 1 diabetes

Diabetes is a chronic disease characterized by an alteration of glucose metabolism, resulting in hyperglycemia, due to an absolute or relative deficit of insulin. It mainly includes four types: autoimmune diabetes (type 1 diabetes mellitus - DM1 or latent autoimmune diabetes in adults - LADA), which involves the destruction of the β-cells of the pancreas with consequent absolute insulin insufficiency, type 2 diabetes mellitus (DM2), more frequently associated with insulin resistance and relative insulin deficiency, gestational diabetes mellitus (GDM), a transient form of glucose intolerance first recognized during pregnancy and monogenic diabetes, a heterogeneous group of disorders caused by mutations in single genes crucial for β-cell function, insulin production, or insulin action. The Research Topic “*Diabetes, transplantation and regenerative medicine*” focuses on key aspects of diabetes and transplantation. It addressed the need to enhance the evidence on the pathogenetic/therapeutic pathways of metabolic alterations secondary to solid organ transplantation (SOT), as well as innovative therapies for the treatment of diabetes relating to β-cell replacement and regenerative medicine.

Conventional therapies for diabetes focus on exogenous insulin administration for DM1 and the use of lifestyle modification coupled with drugs that improve insulin secretion/sensitivity or reduce blood sugar via other mechanisms for DM2. However, these strategies are not curative and may not be effective in preventing long-term complications. Yang et al. performed a bibliometric analysis that highlighted a more dynamic approach towards interventions aimed at diabetes remission, underlining the evolution of therapeutic strategies. In this context, interventions such as bariatric surgery, pharmacological treatment and lifestyle modification are associated with a growing interest in transplant, cell therapies and regenerative medicine.

Cell replacement alternatives offer potential curative approaches. The main β-cell replacement strategies include pancreas transplantation (PTx) and islet transplantation (ITx). PTx is mainly indicated for patients with DM1 and consists of the transplant of an entire pancreas from a deceased donor. It is often combined with kidney transplant in cases of concomitant end-stage renal failure. This procedure can restore normoglycemia and insulin independence. However, it involves a major surgery which is not free from important post-surgical complications and requires the use of lifelong immunosuppressive therapy (IS) to prevent rejection. ITx involves isolating pancreatic islets from a deceased donor pancreas and, in its standard form, infusing them into the portal vein to allow implantation in the recipient’s liver and subsequently promoting glucose- and nutrient-stimulated insulin secretion. This procedure effectively eliminates severe hypoglycemia and may normalize glycemic control with resulting cessation of exogenous insulin therapy or reduce exogenous insulin requirements while maintaining adequate glycemic control in patients with DM1 but lifelong IS is also needed. New pharmacological approaches to IS have contributed to improve patient and graft survival rates. However, current maintenance IS regimens rely on the use of calcineurin inhibitors (i.e. tacrolimus) which have diabetogenic and nephrotoxic effects. Thus, inducing tolerance to the allograft would represent a significant advantage in reducing or mitigating complications related to IS. Q. Chen et al., in their *in vivo* and *in vitro* study on liver transplantation, demonstrated that Hepatocyte Growth Factor (HGF) exerts immunoregulatory effects primarily by suppressing allogeneic CD8^+^ T cells through FAS-mediated apoptotic pathways. Their work introduces the possibility of new therapeutic options to decrease or eliminate the need for chronic immunosuppression, which should be further explored.

Beyond the allogeneic responses after transplantation, recurrence of autoimmunity remains an understudied issue in the field of β-cell replacement therapies. Joana Lemos et al. present a retrospective study on a cohort of 47 ITx recipients transplanted from 2000 to 2018. The authors found that persistent negativity for anti GAD65 and/or IA2 autoantibodies is associated with longer allograft survival, emphasizing the concept that autoimmunity appears to play a role in the pathogenesis of islet allograft dysfunction (Lemos et al.).

In the context of ITx, a factor that limits the amount of engrafted islet mass via the intrahepatic approach is the triggering of an instant blood-mediated inflammatory reaction as the islets come into contact with blood. This reaction has been shown to lead to a substantial loss of transplanted islets. Zhou et al. provide an extensive review on regenerative medicine techniques aimed at improving graft survival rates and enhance the feasibility and efficacy of ITx. In their review, clinically feasible extrahepatic transplantation sites are discussed, with a particular focus in the subcutaneous site, the co-transplantation of islets with other cells or with growth factors, or the use of microencapsulation to reduce inflammation, stimulate angiogenesis, decrease immune activity against the graft, and maximize survival of the islets.

Regarding SOT, there is a high incidence of metabolic disorders in SOT recipients. Most of these alterations are indeed related to chronic immunosuppression. While post-transplant diabetes mellitus (PTDM) and disorders of glucose metabolism are a major concern due to the use of glucocorticoids, osteopenia and osteoporosis also occur frequently. H. Chen et al. investigated, in a retrospective cohort study, the risk of osteoporosis and related fractures in recipients of SOT. The results showed that SOT recipients have a higher risk of osteoporosis and pathological fractures compared to the general population, with the highest risk observed in patients receiving heart or lung transplants.

The relative β-cell deficiency that is secondary to the use of IS (particularly calcineurin inhibitors such as tacrolimus) is another contributor to the development of PTDM, the most frequent metabolic complication after transplantation. The risk factors for PTDM largely overlap with those for DM2. Among the non-common factors, there are peculiar characteristics of the donor, the type of transplant procedure, and the IS regimen, with conflicting data on the type of IS induction. This Research Topic introduces a report by Gupta et al. where the authors examined the impact of IS induction on PTDM in a propensity-matched cohort of heart transplant patients, finding no significant correlation between the use or type of IS induction and the development of PTDM.

PTDM in more fragile patients with frequent episodes of rejection and variable clinical conditions is difficult to manage and imposes an additional burden on the patient due to hyper- and hypoglycemic events and glycemic variability, which negatively impact quality of life. Further, solid organ recipients with pre-existing DM2 experience deterioration in glycemic control following transplantation requiring optimization of their diabetes regimen which may include additional oral anti-hyperglycemic agents and/or introduction of insulin therapy. Many factors such as adherence to therapy, lifestyle, and psychosocial conditions can influence glycemic variability in patients with diabetes. Gan et al. present an intriguing retrospective study including 369 DM2 patients, where factors affecting the variability of glycated hemoglobin (A1c) were evaluated, revealing that baseline A1c, lipids, and uric acid are among the most impactful. This information may serve as a guideline to develop strategies to prevent glycemic variability in transplant recipients with DM2 or in those who develop PTDM.

During IS induction or treatment of rejection episodes, when high doses of immunosuppressants are used, management of PTDM consists almost exclusively of insulin therapy. However, other medications commonly used in DM2 can also be employed in this population when clinical stability is achieved. Notably, innovative pharmacological agents such as glucagon like peptide-1 (GLP-1) receptor agonists, dual GLP-1/GIP agonists, and sodium-glucose co- transporter 2 (SGLT2) inhibitors may be particularly well-suited for use in PTDM. These medications closely correspond to the fundamental mechanisms of PTDM and their glycemic and extraglycemic effects, including cardiovascular and renal protection, and could provide significant benefit in this population as well. Although the use of SGLT2 inhibitors in transplant patients has raised safety concerns due to an increased risk of genitourinary infections and the possibility of euglycemic diabetic ketoacidosis, recent evidence regarding the risk in SOT patients compared to the DM2 population is reassuring. As part of this Research Topic, Fujiwara et al. reported their positive experience with the use of an SGLT2 inhibitor in cancer patients, a population that shares many similarities with SOT in terms of fragility, exposure to immune system modulation therapies, and a higher risk of infections and metabolic side effects including development of diabetes.

The sustained rise in organ transplant procedures, coupled with advancements in surgical techniques and IS protocols, is poised to escalate the occurrence of metabolic complications following transplants, such as PTDM. Studies are needed to evaluate new therapies associated with a lower risk of metabolic disorders, infections, and malignancies. From a diabetes perspective, immunosuppressive agents without associated β-cell toxicity are urgently needed in transplant medicine to largely reduce or eliminate post-transplant dysglycemia and PTDM and clinical trials on the transplant population should be a priority to assess the safety and efficacy of innovative therapies for diabetes prevention and control. Ongoing research endeavors explore advanced therapies and technologies in the quest for a diabetes cure. The utilization of cell therapies remains an increasingly intriguing avenue, showing immense promise as it undergoes continual exploration and refinement in the field.

## Author contributions

AM: Conceptualization, Data curation, Formal Analysis, Funding acquisition, Investigation, Methodology, Project administration, Resources, Software, Supervision, Validation, Visualization, Writing – original draft, Writing – review & editing. DB: Writing – original draft, Writing – review & editing. BM-G: Writing – original draft, Writing – review & editing. MO: Writing – original draft, Writing – review & editing. VS: Writing – original draft, Writing – review & editing.

